# Our Voluntary Hospitals: Some General Effects of the War

**Published:** 1916-01-15

**Authors:** 


### OUR VOLUNTARY HOSPITALS.

### Some General Effects of the War.

Whoever has studied the psychology of markets is familiar with that phase when stocks and shares, or wholesale dealings in commodities, are generally good "in sympathy" with sections that have advanced prices for a material reason. Again, in retail dealings the fact that competition is good for all is often proved by the existence of shops selling the same articles carrying on a prosperous trade not merely in the same neighbourhood but actually side by side. When people are in a buying mood, competition by those who know how to compete apparently does good to a group.

Business and charity in their symptoms and methods of working closely resemble each other in many ways, and the business man likes to see charity carried on in a businesslike way; he welcomes evidence of principles he is accustomed to apply to his own conduct being applied to everything in which he takes an interest. And he, who provides such a large share of the motive power of the normal volume of charity in this country, will not allow, for lack of his own support, the established charitable institutions of the land to repine because temporarily a heavy call upon his generosity is made by the war charities. What the business man feels in this respect is, we think, also in the mind of the public at large. Not only is it the general wish, plainly evidenced in many directions, that the normal flow of almsgiving shall not decline, but in addition it appears to be intended that our time-tried institutions shall prosper to a degree that few had dared to hope possible under the circumstances.

### Purse-Strings Unloosened.

It is too early yet to speak very definitely of the financial condition of the individual voluntary hospital, but it is safe to say in respect of general funds, pursuing the function of distributing grants to hospitals, that the financial position is very hopeful. Our analogy of sympathy amongst similar undertakings may apply here. Purse-strings are unloosed as they have perhaps never been before. We see that King Edward's Hospital Fund for London is in a position to distribute as much this Christmas as last, the amount given through the Metropolitan Sunday Fund in 1915 was actually £12,629 more than in 1914, the marvellous growth of the League of Mercy has not been in any way impeded, and the Hospital Saturday Fund's receipts were stated in a recent report to be £4,000 in advance of the corresponding period of 1914. These figures, of course, apply only to London, but in other great centres of industry, such as Manchester, Birmingham, Liverpool, Sheffield, Cardiff, Belfast, or Glasgow, it is probable that similar conditions prevail. Indeed, from several widely separated places we have assurance that this is the case.

In dealing with this question we do not fall into the error of concluding that increased income necessarily means prosperity. The purchasing power of money is not more when it is in the hands of hospitals than when in the possession of individuals. Indeed, in some important instances hospitals have to buy certain goods the important increase in price of which hardly touches the ordinary man at all. We have a case in mind where every rise of a shilling a pound in the price of a certain drug makes a difference to the annual dispensary expenditure of £160, and as the article in question has advanced from 1s. 6d. to 22s. per pound during the war, the strain on the resources of this particular institution may easily be calculated.

In answer to the question "How is the war affecting your hospital?" the answer is fairly unanimous: "People are very good, funds are coming in well, but see what we have to do with the money." Yet at the worst it is a great thing that people are very good and that they are still in the position to give material proof of their goodness.

### Some Examples.

But as regards expenditure, all institutions are not equally badly hit; the instance given above applies to the peculiar needs of a special hospital. In respect of the Neath Hospital at Dublin, on the other hand, we learn that the ordinary expenditure of this institution, with 164 beds occupied on the average for the first nine months of 1915, was only £220 in excess of the corresponding period in 1914. At the Royal Infirmary, Sheffield, with an occupancy of 274 against 280 there was an actual decrease in expenditure, apart from salaries, of over £500, and this is all the more unexpected as the period January 1 to September 30, 1914, was governed by practically normal conditions. This hospital is receiving wounded soldiers, and has not accepted a Government grant in respect of this service. We are pleased to see that the donation total shows an increase over 1914 of £1,518, and although the subscription receipts as shown by the books were slightly lower on September 30, outstanding subscriptions have since been paid and have made good this decrease.

Carrying our investigations to a far-distant institution—the Royal Infirmary, Aberdeen—we find another instance of generous support on the part of the public. In consequence of a heavy deficit at the end of 1914, a special appeal was made for donations and new annual subscriptions. The result was most gratifying, and one result is that the annual subscription list for the first nine months of 1915 was over £400 ahead of the same period in 1914. The prices of provisions, etc., have, of course, been higher, but it would be misleading to make a comparison of the gross ordinary expenditure, because in 1914 very large sums were spent in replenishing stores in several departments and in renewing certain plant. The hospital has received an unusually large number of contributions in kind, including highly-prized allocations from consignments sent by the people of Queensland through the Agent-General in this country. Up to October 31,

1915, from the beginning of the war, 392 soldiers and 161 sailors have passed through the wards, and, in addition, a considerable amount of work has been done in the electrical department for soldiers sent from the 1st Scottish General Hospital, which military institution is officered by seventeen members of the honorary medical and surgical staff of the Royal Infirmary and has for principal matron the matron of the same institution.

To return to London and to select a representative hospital—Westminster; we find a record which is aptly described by the authorities as "not altogether unsatisfactory." The average occupancy of beds remains steady, and those used for soldiers are fifty-eight of the seventy-five offered. During the nine months under review there was an increase of £326 in ordinary expenditure as compared with the previous year. Under Surgery and Dispensary, the cost actually declined by £167, this probably being due to the fact that the out-patient department showed the important decrease of 20 per cent. in the number of individual patients registered up to December 30, 1915, as compared with the corresponding period of 1914. But taking casualties separately, it is expected that the year's figures will show no corresponding diminution. In this respect the significant remark is made that "it is rather a sign of the times that the number of fatal accidents brought to the hospital at night in the month of September was two and in October six."

In respect of the financial aspect of the general effects of the war upon the voluntary hospitals, it is unsafe to generalise on insufficient data. From the point of view of income, we have noticed many hopeful signs, but we do not doubt that the claims of some institutions, the useful work of which has not in the clamour of national events come prominently before the public, have been unavoidably neglected. It will be our duty, then, with more minute analysis, to return to this subject in the near future.

